# Influence of Naturally Occurring Simian Foamy Viruses (SFVs) on SIV Disease Progression in the Rhesus Macaque (*Macaca mulatta*) Model

**DOI:** 10.3390/v5061414

**Published:** 2013-06-06

**Authors:** Anil Choudhary, Teresa A. Galvin, Dhanya K. Williams, Joel Beren, Mark A. Bryant, Arifa S. Khan

**Affiliations:** 1 Laboratory of Retroviruses, Division of Viral Products, Center for Biologics Evaluation and Research, US Food and Drug Administration, Bethesda, MD 20892, USA; E-Mails: anil.choudhary@fda.hhs.gov (A.C.); teresa.galvin@fda.hhs.gov (T.A.G.); dhanya.williams@fda.hhs.gov (D.K.W.); 2 Division of Veterinary Services, Center for Biologics Evaluation and Research, US Food and Drug Administration, Bethesda, MD 20892, USA; E-Mail: joel.beren@fda.hhs.gov; 3 Division of Veterinary Resources, Office of Research Services, National Institutes of Health, Bethesda, MD 20892, USA; E-Mail: bryantm@ors.od.nih.gov

**Keywords:** Indian rhesus macaques, simian immunodeficiency virus, simian foamy virus, SIV pathogenesis, preclinical AIDS model, dual retrovirus infections

## Abstract

We have investigated the influence of naturally occurring simian foamy viruses (SFVs) on simian immunodeficiency virus (SIV) infection and disease in Indian rhesus macaques. Animals were divided into two groups based upon presence or absence of SFV; in each group, eight monkeys were injected with SIV_mac239_ virus obtained from a molecular clone and four were injected with medium. Blood was collected every two weeks for evaluation of SIV infection based upon T cell-subsets, plasma viral load, development and persistence of virus-specific antibodies, and clinical changes by physical examination and hematology. Comparative analysis of SFV+/SIV+ and SFV−/SIV+ monkey groups indicated statistically significant differences in the plasma viral load between 6–28 weeks, particularly after reaching plateau at 20–28 weeks, in the CD4^+^ and CD8^+^ T-cell numbers over the entire study period (2–43 weeks), and in the survival rates evaluated at 49 weeks. There was an increase in the plasma viral load, a decreasing trend in the CD4^+^ T cells, and a greater number of animal deaths in the SFV+/SIV+ group. The results, although based upon a small number of animals, indicated that pre-existing SFV infection can influence SIV infection and disease outcome in the rhesus macaque model. The study highlights consideration of the SFV status in evaluating results from SIV pathogenesis and vaccine challenge studies in monkeys and indicates the potential use of the SFV/SIV monkey model to study the dynamics of SFV and HIV-1 dual infections, recently reported in humans.

## 1. Introduction

Various simian immunodeficiency viruses (SIVs) and chimeric simian-human immunodeficiency viruses (SHIVs) have been used in different macaque species for studying AIDS pathogenesis and evaluation of vaccine efficacy [1]. A well-established model for investigating the correlates of protection and studying SIV infection and disease progression is SIV_mac239_ infection in Indian rhesus macaques. A major challenge in the nonhuman primate (NHP) models has been variability of results, even when using the same inoculum. This may be due to various host factors including genetic, immunologic, microbiologic, and age-related, or to environmental factors such as housing and handling, which can induce stress or introduce exogenous viruses. An important virologic factor that is generally overlooked is the high prevalence (75%–100%) of simian foamy viruses (SFVs) in nonhuman primates, which can become 90%–100% during captivity. SFV infection in monkeys is generally latent; although proviral DNA can be detected in all tissues, viral RNA expression is seen only in oral tissues [2,3]. Circulating SFVs have coevolved with their host species [4] and the virus genome diversity seen in naturally occurring viruses may have occurred due to intra-species or inter-species transmission [5]. 

SFV has a very broad host range and tissue tropism. Although SFV can result in a lytic infection in fibroblasts in tissue culture and a persistent infection in epithelial cells [6], there is no evidence of clinical signs *in vivo* in any animal species by natural or experimental infection [7,8]. SFV infections have also occurred in humans due to occupational exposure to infected NHPs or tissues or in natural settings [9,10,11,12,13,14] and can result in lifelong persistence of infectious virus [15]. In fact, SFV and HIV-1 coinfections have been reported in Africa [16]. Although there is no evidence of SFV-induced disease or human to human transmission, dual retrovirus infections may pose a concern based upon results from a retrospective study that showed expanded tissue targets of SFV expression were seen in SIV-immunosuppressed monkeys [17]. This concern is particularly relevant to SFV-infected populations in Southeast Asia and Central Africa, which are also high risk areas for HIV-1 infections. Furthermore, *in vitro* experiments and transgenic mouse studies have shown that prototype foamy virus (originally designated as human foamy virus) can increase HIV-1 gene expression [18,19] and cell binding [20]. Our study was designed to investigate the influence of naturally occurring SFV infections on SIV-induced AIDS in rhesus macaques and to evaluate the potential use of the SFV/SIV monkey model for investigating consequences of dual SFV and HIV-1 infections in humans. 

## 2. Results and Discussion

### 2.1. Study Design

Dual SFV and SIV infection was investigated in the well-established SIV_mac239_-Indian rhesus macaque model [1]. Monkeys selected for this study were initially evaluated clinically for good health (body weight, CBC differential, and serum chemistry) and prescreened for simian viruses and other microbial agents. All animals were individually housed during this screening period and thereafter. Immunophenotyping was done to determine the number of CD4^+^ T cells, CD8^+^ T cells and B cells. MHC class I and class II genes that have been reported to influence SIV infection [21,22] were determined retrospectively. Additionally, to minimize variability in the results due to genetic diversity in the virus inoculum, SIV_mac239_ virus stock was prepared from an infectious, cloned DNA [23] with minimum cell passage. The details are described in Section 3.1. 

**Figure 1:**
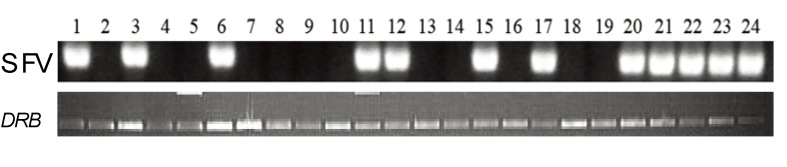
SFV status in monkeys by PCR analysis. Peripheral blood mononuclear cell (PBMC) DNA was isolated from 24 monkeys at 5 months prior to initiation of the study and analyzed by a nested PCR assay using SFV set B primers resulting in a 390 bp fragment. PCR amplification of a ~300 bp *Mamu-DRB* gene fragment was used as a control to determine the presence of intact DNA in the test samples.

Although 24 animals were initially selected as SFV negative based upon serological screening of 70 animals, subsequent PCR analysis indicated that only 12 animals were SFV negative and 12 were SFV positive. The results are shown in Figure 1. Virus identity was confirmed by nucleotide sequence analysis of the PCR amplified fragments. The SFV status in the animals was confirmed at various time points prior to study initiation.

Based upon the SFV status of the animals, monkeys were divided into two groups consisting of 12 SFV-negative animals and 12 SFV-positive animals (designated as SFV− and SFV+, respectively): in each group, eight animals were injected with SIV_mac239_ (1000 TCID_50_ or 12.33 ng p27 antigen per mL per animal) and four were injected with medium as controls. Monkeys that were selected for SIV inoculation had good health and a normal range of CD4^+^ and CD8^+^ T-cell counts. 

### 2.2. Evaluation of T and B cells

**Figure 2:**
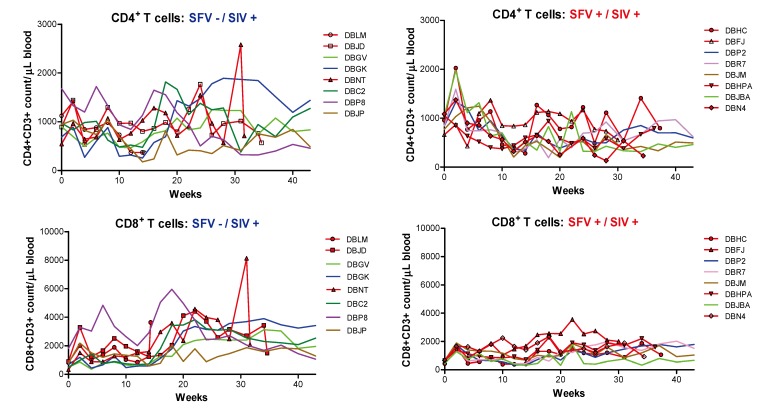
Comparison of CD4^+^ and CD8^+^ T cells in SIV-infected monkeys. The numbers of CD4^+^ T cells and CD8^+^ T cells were determined for week 0 (prior to SIV injection) and after injection through week 43 for each monkey or earlier in case of termination due to disease (indicated in red with a symbol). Animals are grouped based upon SFV status for comparison and listed individually in each group.

SIV_mac239_ infection in rhesus macaques results in decreasing CD4^+^ T-cell numbers with disease progression, and quantitative and qualitative changes in the CD8^+^ T cells occur in response against the infection. Therefore, CD4^+^ and CD8^+^ T cells were monitored in this study to compare these parameters in SIV-infected, SFV negative and SFV positive monkeys. The absolute number of T cells was calculated using the percentage of CD3^+^ T-lymphocyte subsets (CD4^+^, CD8^+^ cells) determined by flow cytometry, and the number of total lymphocytes was determined from the CBC analysis. The T-cell data for individual animals are shown in Figure 2. There was a decrease in the CD4^+^ T cells through week 14 seen in all of the animals in the study including controls (data not shown), which may be age-related, followed by recovery in all animals, except those in the SFV+/SIV+ group. Furthermore, increases in the CD8^+^ T cells were seen in the SFV-/SIV+ monkeys after week 14 but not in the SFV+/SIV+ animals.

**Figure 3:**
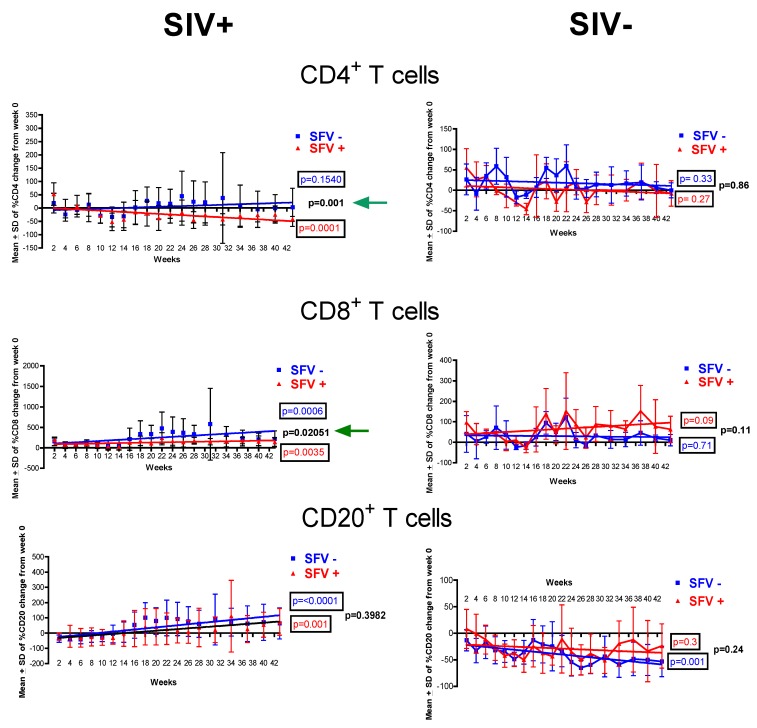
Longitudinal analysis of T and B cells. Linear regression (GraphPad Prism, v5.0, La Jolla, CA, USA) of percent changes in T and B cells in SFV positive and SFV negative monkey groups was done by calculating the percent change in cells as compared to week 0 for each animal at each time point. CD4^+^ and CD8^+^ T cell counts and CD20^+^ B cell counts were determined by flow cytometry using freshly collected blood in EDTA anticoagulant. The groups of SIV positive and control SIV negative monkeys are shown. Mean ± SD was determined based upon the percent change in cells for all of the monkeys at each time point per group. The difference in the slope of the linear regression curve with regard to week 0 is indicated by the boxed *p* values. The statistically significant *p* values between the monkey groups are indicated by an arrow (<0.1).

To further evaluate the changes in the T-cell subsets, the results were longitudinally evaluated based upon a linear regression analysis of all of the animals in each group (8 in each of the test groups: SFV−/SIV+, and SFV+/SIV+; 4 in each of the control groups: SFV−/SIV−, and SFV+/SIV−) by calculating the percent change in the number of cells in each animal at each time point, after normalizing to week 0 (Figure 3). The results showed a decreasing trend in the CD4^+^ T cell number in the SFV+/SIV+ animals as compared with the SFV−/SIV+ animals (*p* = 0.001). In contrast, a trend toward an increase in the CD8^+^ T cells was seen in these two animal groups; however, a lesser increase was seen in the SFV+/SIV+ animals as compared with the SFV−/SIV+ animals (*p =* 0.02051). Similar results were obtained when the data was re-analyzed without DBNT and DBGK, assuring that some of the elevated T-cell numbers associated with these two animals did not skew the trend analysis in the SFV−/SIV+ group of animals. An increasing trend was seen in the CD20^+^ B cells in the SIV-infected animals, but the difference between the SFV positive and SFV negative groups was not statistically significant (*p =* 0.1). Furthermore, there was no statistical difference in the T-cell subsets or B cells between the SFV positive and SFV negative animals in the control SIV negative group. These results indicated that SFV may influence T-cell subsets in SIV-infected macaques. 

### 2.3. Plasma Viral Load Analysis in SIV-infected Monkeys

**Figure 4:**
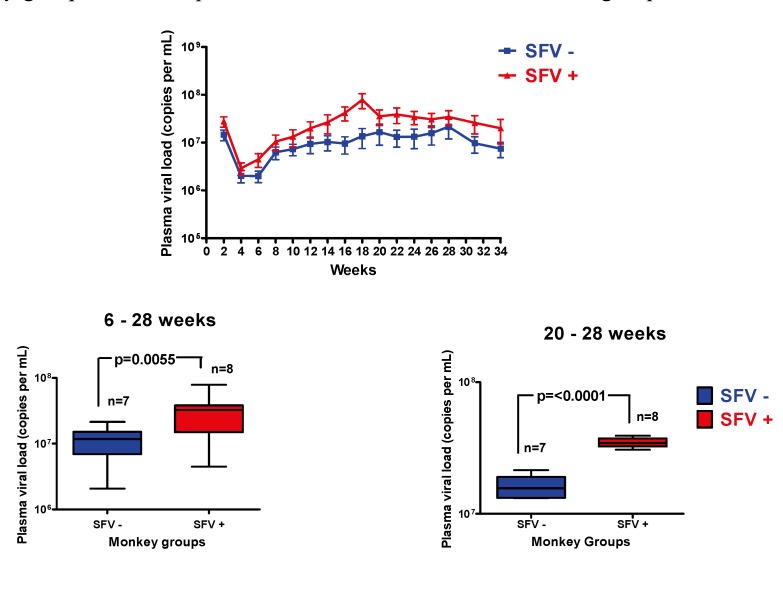
SIV viral load analysis. Plasma viral load was determined at the indicated times by bDNA from weeks 2 through 34. Mean ± SEM of the results from SFV+/SIV+ and SFV−/SIV+ monkey groups was plotted 2–34 weeks. Box and whiskers analysis of the plasma viral load from 6–28 weeks and 20–28 weeks for the SFV positive and SFV negative monkey groups is shown; *p* values and number of animals in each group are indicated.

SIV viral RNA load was evaluated by bDNA analysis. The results (Figure 4) indicated an early acute phase and the viral load reached a plateau at about week 20. SIV viral loads were similar to those reported in previous studies [24]. The SFV negative and SFV positive groups had similar viral loads up to week 4, after which the viral load in the SFV positive group was slightly elevated compared with the SFV negative group. A statistically higher viral load was seen in the SFV positive group compared with the SFV negative group between weeks 6–28 (*p* = 0.0055); this was more pronounced at weeks 20–28 (*p* < 0.0001) (Figure 4). The results indicated that pre-existing SFV infection correlated with a higher viral load in SIV-infected monkeys that was maintained over the study period. 

### 2.4. Survival Analysis and Disease Progression

**Figure 5:**
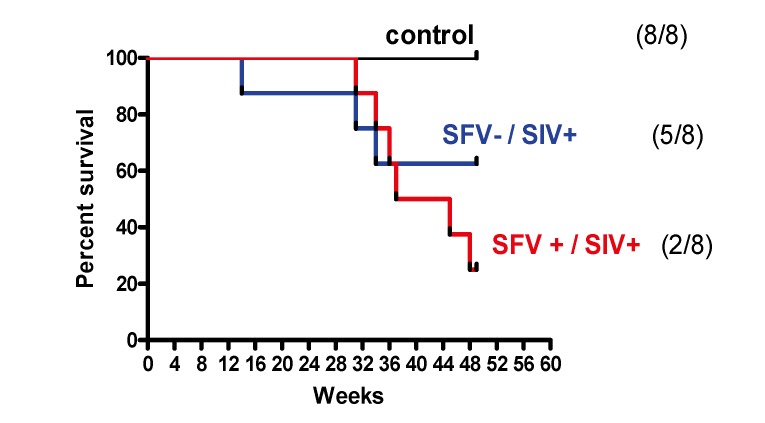
Monkey survival curves. The Log-rank (Mantel-Cox) test indicated the difference in the survival curves as *p* = 0.0217 at 49 weeks after injection.

The influence of SFV infection on SIV clinical outcome was examined by plotting survival curves of the different animal groups. The results (Figure 5) showed 100% survival in the control SIV negative group (which included both SFV negative and SFV positive animals) and about 63% survival in the SFV−/SIV+ group at 49 weeks; however, only 25% survival was seen in the SFV+/SIV+ group. The log-rank (Mantel-Cox) test indicated that there was a significant difference in the survival curves (*p* = 0.0217) at 49 weeks; a negative impact was seen in the presence of SFV on survival of the SIV-infected animals. Animals were euthanized from 30 to 133 weeks after SIV injection upon development of severe AIDS related symptoms, such as significant weight loss (about 20%), loss of appetite, chronic diarrhea, and pneumonia, except in one case (DBGV). Review of the pathological reports following necropsy showed that all SIV-injected monkeys died due to SIV-related disease, except for DBGV, which was terminated at the end of the study and exhibited no severe symptoms at the time of death.

**Figure 6:**
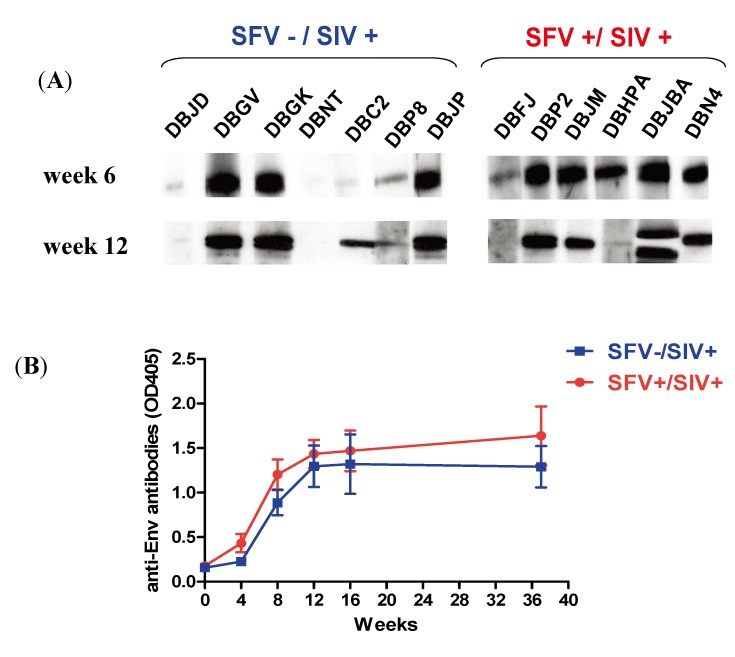
Evaluation of SIV Gag and Env antibodies. (**A**) Plasma samples from week 6 and week 12 were analyzed for Gag antibodies by Western blot assays using p27 antigen. (**B**) Animals that were positive for anti-gp120 antibodies in a Western blot assay using SIV-infected cell lysate were further analyzed by ELISA using 1:100 dilution of plasma and SIV_mac239_ gp120 antigen. The mean ± SEM of animals in the SFV positive group and SFV negative group is shown for selected time points. The number of animals at each time point in the case of the SFV positive group were: five for week 0, 4, 12, and 16 (DBP2, DBR7, DBJM, DBJBA, and DBN4), four for week 8 (no sample was available from DBR7) and 37 (DBN4 died at week 34); and the number of animals in the SFV− group were: six for week 0, 4, 8, and 12 (DBLM, DBGV, DBGK, DBC2, DBP8, and DBJP) , and five for week 16 and 37 (DBLM died at week 14). Data for week 4 and week 37 or terminal time point is shown in Table 1.

The study animals were further grouped based upon the rate of SIV disease progression and SFV status (Table 1). The results showed that one animal that was a rapid progressor (DBLM) was SFV negative, 10 were conventional progressors, of which 3 were SFV negative, and 5 were slow progressors of which 4 were SFV negative. Additionally, the development of p27 Gag antibodies was evaluated at various time points throughout the study period (Figure 6A). A loss of Gag antibodies was seen in some of the animals as expected with development of end-stage AIDS. Furthermore, Env gp120 binding antibodies (Figure 6B) and SIV_mac239_ neutralizing antibodies were evaluated from weeks 0-49 in animals that maintained Gag antibody. Neutralization (ID_50_) was determined in the TZM-bl assay using SIVmac239Cs.23 [25]. The results at week 37 indicated four animals were positive based upon a cut-off value >1:40 (due to assay background at 1:20), which represented the sample dilution at which relative luminescence units (RLUs) were reduced 50% compared to virus control wells (no test sample). These were: DBR7, 1:75; DBJP, 1:179; DBGV, 1:41; and DBP2, 1:518. The results indicated that all the animals were positive for Gag antibodies at 4–6 weeks after SIV injection, and in most cases, thereafter until study termination. In general, there seemed to be higher Gag antibody responses in the SFV positive monkeys, but there were outliers in each group, so no direct correlation could be made between the SFV status, disease progression, and antibodies against SIV based upon the current data (Table 1).

**Table 1:** SIV groups based upon disease progression. Monkeys were grouped based upon rapid, conventional, or slow disease progression. The SFV status and the gp120 ELISA OD are also indicated.

Monkey ID	SFV status	Disease progression (termination week)			Gp120 ELISA OD ^a^ (week 4 and week 37 or earlier terminal week)
		Rapid (<6 month)	Conventional (6–24 month)	Slow (>2 year)	
DBLM	−	14			0.270/1.962
DBFJ	+		30		NT ^b^
DBNT	−		31		NT ^b^
DBJD	−		34		NT ^b^
DBN4	+		34		0.158/0.835
DBHPA	+		36		NT ^b^
DBHC	+		36		NT ^b^
DBR7	+		45		0.235/1.294
DBJM	+		48		0.465/1.983
DBJBA	+		83		0.646/2.367
DBJP	−		83		0.356/1.475
DBP8	−			115	0.167/1.049
DBGK	−			124	0.222/1.136
DBC2	−			133	0.181/0.706
DBGV^c^	−			141	0.159/2.082
DBP2	+			128	0.658/0.864

^a^ Samples were screened at 1:100 dilution in duplicate using SIV239 gp120 antigen; ^b^ NT, not tested due to absence of detectable gp120 antibodies based upon Western blot analysis; ^c^ Euthanized at study termination without clinical symptoms.

### 2.5. MHC Alleles

Certain *Macaca mulatta* (*Mamu*) MHC class I alleles have been associated with protection against SIV infection in Indian rhesus macaques [26]. Animals positive for Mamu-A1*01, Mamu-B*08, and Mamu-B*17 tend to show lower set point plasma viral loads in SIV infection resulting in slower disease progression [27,28,29,30] (indicated in red in Table 2). Mamu-B*17, and Mamu-B*08 have been correlated with spontaneous SIV control; Mamu-A*01^+^ monkeys are long-term nonprogressors (LTNP) and Mamu-A*02^+^ monkeys are intermediate progressors with moderate resistance to SIV infection. Other alleles such as Mamu-A1*02, Mamu-A1*08, Mamu-A1*011, Mamu-B*01, Mamu-B*03, and Mamu-B*04 have been implicated in the restriction of SIV or SHIV CD8^+^ T-cell epitopes. Additionally, Mamu-A*07 may have a potential role in the immune response against SIV [31]. MHC class II alleles Mamu-DRB1*0306 and Mamu-DRB1*1003 have been shown to be highly expressed in elite controllers and to contribute to reducing SIV viral load along with Mamu-B*17-positive animals [21]. To determine if the MHC class I and class II alleles previously studied in SIV infection were involved in the increased in the number of deaths seen in our study in the SFV+ monkeys as compared to the SFV− monkeys, monkey DNAs were submitted for a retrospective study using allele-specific PCR assays [22]. The results are shown in Table 2. It should be noted that the one SFV^+^ SIV^+^ monkey that was a slow progressor (DBP2) had both SIV-controlling alleles Mamu-B*08 and Mamu-B*17 and also had Mamu-DRB1*1003. However, there was no other correlation noted with disease progression and the presence of any of the other alleles tested in this study. All were negative for Mamu-A1*011, Mamu-A1*07, Mamu-B*03, Mamu-B*04, DRB1*0306.

**Table 2:** MHC genotyping of SIV-infected monkeys ^a^.

Monkey ID	SFV status/Monkey survival (weeks)	Class I ^b^		Class II ^b^	
		*Mamu-A*	*Mamu-B*	*Mamu-DR*	*Mamu-DP*
		A*01^c^ A*02 A*08	B*01 B*08 B*17	B1*0306, B1*1003 B*w201, B*w606 B1*04	B1*06
DBLM	−/14	A*02		B*w201, B*w606	
DBFJ	+/30	A*02		B1*0306, B1*1003 B*w606	
DBNT	−/31	A*01 A*08	B*01	B1*1003 B*w201, B*w606	
DBJD	−/34	A*02 A*08		B*w201 B1*04	
DBN4	+/34		B*01	B1*1003 B1*04	B1*06
DBHPA	+/36	A*02	B*01	B1*1003	
DBHC	+/36		B*01	B1*1003 B*w201 B1*04	
DBR7	+/45			B1*0306 B1*1003	
DBJM	+/48	A*02		B*w606 B1*04	
DBJBA	+/83	A*08		B1*1003	
DBJP	−/83			B1*1003	
DBP8	−/115	A*02		B*w201, B*w606	
DBGK	−/124	A*01	B*01	B1*0306, B1*1003 B*w201	
DBC2	−/133	A*02		B1*1003 B*w201, B*w606	
DBGV	−/141	A*02 A*08		B*w201, B*w606	
DBP2	+/128		B*08 B*17	B1*1003 B*w201	

^a^ All were negative for Mamu-A*07, Mamu-B*03 and Mamu-B*04; ^b^ Common allele names are used; ^c ^ Protective alleles are indicated in red.

## 3. Experimental

### 3.1. Animals, Prescreening for Selection, and Management

Indian rhesus monkeys (*Macaca mulatta*) were obtained from a domestic breeding colony in Morgan Island, South Carolina (Alpha Genesis Inc., Yemassee, SC, USA). Monkeys (males; 1.5–2 years old) were initially selected based upon good physical health (with normal body weights between 2–3 kg and no signs of clinical problems) and negative serology for simian pathogens (measles virus, herpes B virus, simian type D retrovirus [SRV], simian T-lymphotropic virus [STLV], and SIV; VRL Laboratories, San Antonio, TX, USA). Additionally, the animals were pre-screened for SFV using a dot blot immunoassay [32] (VRL Laboratories). Animals were de-wormed for potential parasites using ivermectin, droncit, and fenbendazole treatment for 3 days. 

Animals were individually maintained during the pre-screening period, shipping to FDA facilities (National Institutes of Health, Bethesda, MD, USA), and thereafter until the end of the study period. Animals care was in accordance with the *Guide for the Care and Use of Laboratory Animals* [33] under a protocol approved by the Institutional Animal Care and Use Committee (CBER, FDA). The animals were quarantined for 10-weeks upon arriving at the FDA nonhuman primate facilities (NIH, Bethesda, MD, USA): after the initial 2-week acclimation period, animals received complete physical examinations, five intradermal tuberculin tests to assure absence of any *Mycobacteria*. Fecal cultures for bacteriology and parisitology were obtained. Some animals were de-wormed again. Additionally, each animal was tested to confirm their negative status for measles virus, herpes B virus, SIV, STLV and SRV by serology. The SRV status was verified by a virus-specific PCR assay (Pathogen Detection Laboratory, California National Primate Research Center, University of California Davis, Davis, CA, USA). Additionally, the SFV-seronegative status was evaluated in our laboratory by PCR analysis using Set B primers, which can detect naturally-occurring SFVs in rhesus and pig-tailed macaques [34]. DNA was prepared from monkey PBMCs (Qiagen DNA Blood Mini Kit, Valencia, CA, USA). Primers targeting the highly conserved region of the *Mamu*-*DRB* exon 2 (designated as 5'-MDRB and 3'-MDRB) were used as control [35]. Based upon the results, two study groups were formed containing 12 SFV positive and 12 SFV negative animals: each group contained 8 test animals and 4 control animals. At 8-weeks prior to initiation of the study, the SFV status of the animals was confirmed by PCR and all of the animals received routine measles virus vaccine subcutaneously (0.5 mL; Attenuvax, Merck). After an additional 4 week transient immunosuppression period due to the measles vaccine virus, the SFV status was again verified by PCR assay prior to study initiation. 

The clinical health of the animals was evaluated by hematology and serum chemistry (Antech, Lake Success, NY, USA); animals with CBC values in the normal range were used as test animals. Monkey peripheral blood was additionally evaluated for T-cell and B-cell numbers by immunophenotyping T-cell subsets (CD3^+^, CD4^+^ and CD8^+^ T cells) and CD20^+^ B cells (as described below); monkeys with similar cell counts were included in the study as test animals in an effort to minimize variability in the results subsequent to SIV infection. 

In a retrospective analysis, DNA prepared from whole blood (Qiagen DNA Blood Mini Kit) was submitted for MHC class I and class II typing using rhesus sequence-specific priming (PCR-SSP) [22] (University of Wisconsin AIDS Vaccine Research Laboratory, Madison, WI, USA). Class I alleles included: Mamu-A1*01, -A1*02, -A1*08, -A1*07 and -A1*11 (new nomenclature and allele specificity: Mamu-A1*001:01/001:02/001:03/001:04/001:05, -A1*002:01, -A1*008:01 and -A1*011:01, respectively) and Mamu-B*01, -B*03, -B*04, -B*08, -B*17 (new designations and allele specificity: Mamu-B*001:01:01/001:01:02, -B*003:01/066:01, -B*004:01/105:01/106:01, -B*008:01 and *Mamu-B017:01/017:03*. Class II alleles include: DRB*w201, DRB*w606, DRB1*1003, DRB1*04, DRB1*0306, and DPB1*06 (new nomenclature and allele specificty: Mamu-DRB*w201, -DRB*w606, -DRB1*03:01-03:08/03:10-03:18, -DRB1*04:01/06/11, -DRB1*10:03/06/08, and Mamu-DPB1*07:01, respectively).

### 3.2. Preparation and Characterization of SIV Inoculum

Full-length, infectious SIV_mac239_ SpX cloned DNA [23] (kindly provided by R. Desrosiers; NIH AIDS Reagent Program, catalog number 12249) was transformed into *E. coli* Stbl2 cells (Invitrogen) and a large scale preparation was grown in LB broth containing 25 mg per L ampicillin at 30 °C, and DNA purified using Qiagen Maxi kit. The full-length of the DNA was confirmed by restriction enzyme digestion (*Bgl*II, *Hind*III and *Xho*I). SIV_mac239_ virus stock was prepared as previously described [36]. 293T/17 cells (ATCC, Herndon, VA, USA) were transfected at 20%–25% confluence with SIV_mac239_ SpX cloned DNA (5 µg per 75 cm^2^ flask) using Profection Calcium Phosphate Transfection Kit (Promega, Madison, WI, USA). Supernatant was collected at 72 h post transfection and clarified by low speed centrifugation to remove whole cells. 

The virus stock was characterized based upon its replication kinetics and by determining the total p27 antigen content, viral RNA copy number, and infectious titer. The total p27 antigen content was 685 ng/mL as determined by analyzing serial dilutions of the sample (1:128 to 1:512) according to the manufacturer’s protocol in a SIV_mac251_ p27 antigen ELISA kit (Advanced BioScience Laboratories, Rockville, MD, USA). The infectious titer was determined as 10^4.83^ TCID_50_ per mL [37] based upon inoculating serial 10-fold dilutions in 174×CEM cells [38] (NIH AIDS Research and Reference Reagent Program, NIH, Bethesda, MD; catalogue number 272) and determining p27 in the supernatant on day 7. Additionally, plasma SIV RNA was determined as 10^8.9^ copies per mL by a branched DNA (bDNA) signal amplification assay [39,40] (Siemens). The kinetics of SIV_mac239_ replication were evaluated in 174×CEM cells based upon p27 antigen in the supernatant at days 3, 7, 10, and 13, collected just prior to splitting the cells. The kinetics of virus replication were as expected and a virus peak was seen on day 7 [41]. 

### 3.3. Animal Injections, Blood Collections, and Necropsies

Prior to SIV injection, control samples of blood were collected at various times in both EDTA and heparin anticoagulants for preparation of plasma and PBMCs to use in various assays for evaluation of SIV infection after monkey injection. At the time of virus injection (designated as week 0) and after injection (generally every two weeks), blood was collected in EDTA tubes for CBC differential and immunophenotyping; in EDTA PPT tubes for plasma separation for bDNA analysis (Becton, Dickinson and Company, Franklin Lakes, NJ, USA; catalogue number 362788); and in heparin CPT tubes (BD, catalogue number 362753) for PBMC preparation and plasma collection for ELISA and western blot analysis. Additionally, animals underwent a routine physical examination at time of each bleed. Blood was collected under sedation using ketamine hydrochloride (10 mg/kg). Animals were necropsied after development of severe AIDS-related symptoms (except in case of DBGV, a slow progressor that was necropsied at termination of the study) and tissues were collected for evaluation.

SIV_mac239_ inoculum (1000 TCID_50_ or 12.33 ng p27 antigen per mL per animal) was prepared by diluting the original stock in RPMI medium without added serum or other supplements (Quality Biologicals, Gaithersburg, MD, USA). Control animals received 1 mL medium. 

### 3.4. Flow Cytometry

EDTA-blood was pre-washed with room temperature cell-washing buffer (Dulbecco’s PBS, without Ca^++^ or Mg^++^; Quality Biologicals), and the original volume was reconstituted with the same buffer. Antibodies (10 µL each; FITC anti-CD3 epsilon, anti-CD4-PE, anti-CD8-PerCP and anti-CD20-FITC, catalogue number 556611, 550630, 347314 and 347673, respectively; BD Biosciences, San Jose, CA, USA) were added to 100 µL aliquots of washed blood and incubated for 1 h at room temperature. Red blood cells were then lysed using BD FACS Lysing Solution (BD Biosciences, San Jose, CA, USA) according to the manufacturer’s instructions. Cells were pelleted and washed three times in cell-washing buffer and fixed overnight at 4 °C in 250 µL of 2% paraformaldehyde (EM-grade from Electron Microscopy Sciences). For flow cytometry analysis, ten thousand events were acquired in the lymphocyte region using Cell Quest (v3.1) on a FACSCalibur (Becton Dickinson). Data were analyzed using FlowJo software (v6.1.1; Treestar, Ashland, OR, USA).

### 3.5. Plasma Viral Load

EDTA plasma was evaluated for SIV RNA by a bDNA assay (Siemens, CA, USA). Plasma was aliquoted into single-use vials to avoid freeze thaw of samples. Bayer Reference Testing Laboratory performed viral quantitation of SIV in plasma samples using the Versant branched DNA (bDNA) method. RNA was extracted from 0.5 mL of monkey plasma and combined with probes targeting the *pol* region of SIV to allow for hybridization and binding to the microwell. The probe signal was then amplified to allow for detection of the target SIV RNA. The level of viral RNA in the test sample is quantified by comparing generated signals to a standard curve made using *in vitro* transcribed SIV_mac239_*pol* RNA. The results were reported in copies per mL.

### 3.6. Antibody Detection

Anti-SIV antibodies were estimated in plasma collected in heparin from monkeys injected with SIV_mac239_. Western blots were used to screen for the presence of antibodies in plasma over the period of the entire study. For performing western blots, purified SIV_mac251_ p27 (Advanced Bioscience Laboratories, MD, USA, catalogue number 4530) was run on 10% SDS-PAGE for 1.5 h at 125 V and transferred to nitrocellulose membrane for 1 h at 30 V. The membranes were blocked overnight with PBST+5% non-fat milk, incubated with plasma at 1:100 dilution for 2 h at room temperature and then at 4 °C overnight. The next day membranes were washed 4 times with PBST+5% non-fat milk followed by incubation with anti-monkey-HRP conjugated IgG (Cappel, OH, USA, catalogue number 55432) secondary antibody at 1:500 in PBST+5% non-fat milk, for 2 h at room temperature. After washing 3 times with PBST+5% non-fat milk followed by a 20 min wash with PBST, the membrane proteins were visualized by chemiluminescence using SuperSignal WestPico Chemiluminescent Substrate (Pierce) and exposed on X-ray films.

## 4. Conclusions

The study showed that pre-existing, natural SFV infection significantly influenced the results of experimental SIV infection in the rhesus macaque model; there were increases in SIV plasma viral load, in loss of CD4+ T cells, and in animal deaths as compared to SFV negative animals. The results underscore the importance of SFV screening of monkeys used in SIV pathogenesis and AIDS vaccine studies. Further investigations of dual retrovirus expression in SFV/SIV co-infected animals will provide insight regarding virus-virus and virus-host dynamics and may elucidate the potential role of SFV in enhancing SIV infection and disease. 
